# The TCI Clinical Encounter Program for PhD Students in Cancer Biology: a Feasibility Pilot

**DOI:** 10.1007/s13187-021-02088-x

**Published:** 2021-10-14

**Authors:** Alexander M. Real, Jenny J. Lin, Janice L. Gabrilove

**Affiliations:** 1 The Tisch Cancer Institute, Icahn School of Medicine at Mount Sinai, New York, NY USA; 2 Graduate School of Biomedical Sciences, Icahn School of Medicine at Mount Sinai, New York, NY USA; 3 Department of Medicine, Icahn School of Medicine at Mount Sinai, New York, NY USA

**Keywords:** PhD training, Cancer biology, Clinical exposure

## Abstract

Clinical rotations are often not included in graduate-level cancer biology curricula; however, basic insight into clinical oncology is often crucial for developing translational research that addresses unmet needs with the potential to benefit cancer patients. We describe a needs assessment, design, implementation, and descriptive evaluation of an oncology-specific pilot clinical encounter program developed for PhD students in the Cancer Biology Training Area (CAB) in the Graduate School of Biomedical Sciences (GSBS) and Tisch Cancer Institute (TCI) at the Icahn School of Medicine at Mount Sinai (ISMMS). Prior to the development of this pilot program, CAB students, in years 2–5 + , were surveyed to determine their interest in a structured clinical experience. Seventeen out of thirty-one students responded (55%) to the survey. Of those seventeen respondents, fifteen (88.2%) expressed that exposure to cancer patients in the clinical setting would be useful for their pre-doctoral biomedical science and cancer biology training and indicated an interest in participating in the clinical encounter program. Based on these responses, a three-session clinical encounter pilot program was designed. Two separate cohorts of 5 students participated in this pilot program. During a formal debrief, following the clinical experience, students commented on the resilience of patients and the importance of research on clinical decision making, and reported that they found the experience motivational. Five out of 10 students responded (50%) to a post-program assessment survey; all five respondents answered that they would recommend the clinical encounter program to their peers. While limited in size and scope, this pilot TCI Clinical Encounter Program proved feasible and has the potential to enrich and inform the experience of PhD students pursing advanced degrees in a cancer biology.

**Supplementary Information:**

The online version contains supplementary material available at 10.1007/s13187-021-02088-x.

## Introduction

The ability to translate basic science research discoveries into clinically useful therapies and practices is one of the overarching goals of advanced training in oncological science, but formal programming to immerse basic science PhD students in clinical environments is lacking for many graduate-level cancer biology training programs. A 2017 survey administered to graduate students across the 26 institutions comprising the Cancer Biology Training Consortium (CABTRAC) showed that while students rate clinical rotations and physician shadowing programs highly on a scale from 1 to 5 in importance to their training (median score of 4), less than 30% of survey respondents report having clinical rotations or physician shadowing programs as a component of their PhD training [1]. In contrast, required rotations in research laboratories have been incorporated into the curriculum for medical students prior to graduation in nearly half of all accredited medical schools, to advance insights into relevant biological principles [2]. The absence of structured clinical experiences for many PhD-level students in oncology presents an unmet educational need as well as a rich opportunity for cross-training between the related disciplines of clinical care and basic science.

To address this unmet need, a pilot Clinical Encounter Program was designed for PhD students in the Cancer Biology Training Area (CAB) at the Icahn School of Medicine at Mount Sinai and the Graduate School of Biomedical Sciences. The program leverages a unique feature of the training environment at Mount Sinai, which is the close proximity of the basic science research facilities to the clinical care floors—in some instances even residing in the same building. The aims of this educational initiative were to provide CAB students with clinical exposure, allowing them to (1) gain insight and motivation in their field of research from interactions with patients, (2) learn to communicate their research in lay-friendly language, and (3) gain a better understanding of clinical disease context, diagnostics, manifestations, and treatment plans for the illnesses they study. The aim of our post-program evaluation was to qualitatively and quantitatively explore the impact of this pilot clinical encounter program on these intended outcomes.

## Material and Methods

### Needs Assessment Survey

Prior to the start of the pilot program, all CAB PhD students (year 2 or more of PhD training) at ISMMS were surveyed in the fall of 2019 using Google Forms. Students were asked to indicate their level of interest in participating in the pilot program, the number of years of PhD training they had completed, whether they had prior clinical experience, if they felt a clinical encounter program would be useful for their training in biomedical science, and if they had a specific disease area of interest relevant to their research (full survey in Supplementary Table S1). These questions were designed to determine the level of clinical exposure at baseline and to assess the perceived value of a clinical encounter experience in biomedical graduate education across the years of PhD training. These questions were reviewed by members of the Tisch Cancer Institute (TCI) Education and Training Committee as well as the Co-Director of the CAB training area (Dr, Doris Germain) before administration to the students.

### Program Design

**Fig. 1:**
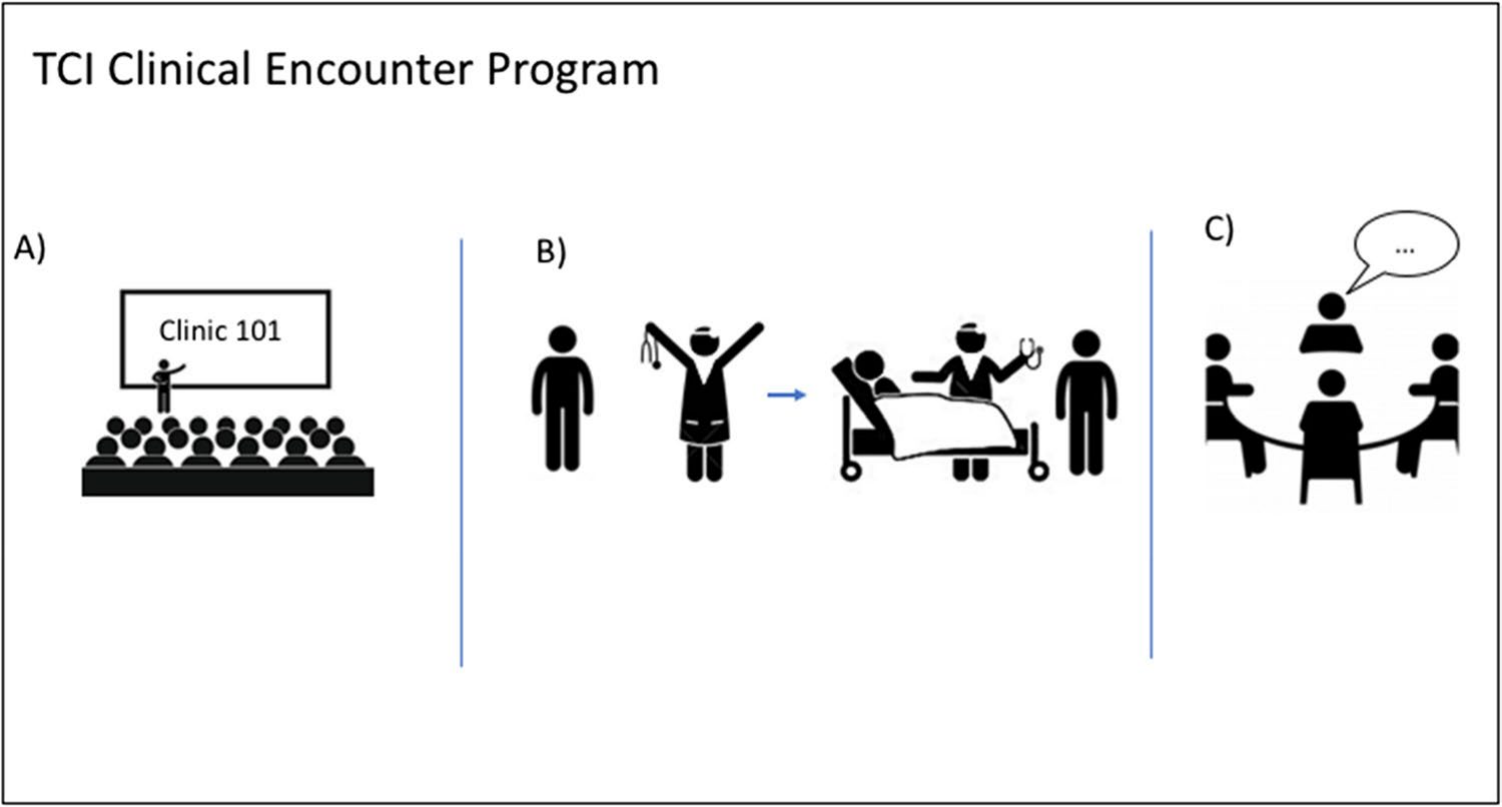
**A** The first session of the TCI Clinical Encounter Program consists of a physician-led lecture on clinical conduct and professionalism, as well as a group exercise practicing a research one-liner. **B** The second session starts with introductions between pilot program participant students and a physician “matched” to their disease area of interest, which leads into clinical encounters facilitated by the physician. **C** The third session consists of a physician-led debrief

The TCI Clinical Encounter Program was designed by the authors in conjunction with the leadership of the CAB, to provide CAB PhD students with insight into core professional attitudes essential for and integration of scientific discovery into clinical oncology. The clinical encounter program for PhD students was divided into three sessions (Fig. 1). The first session consisted of a physician-led discussion on clinical conduct and professionalism, as well as a group exercise where students practiced briefly describing their research in lay terms as a “one-liner” to present to patients or clinicians if asked. This Clinic 101 “Rules of the Road” introductory lecture for PhD students was adapted from the ISMMS “Art and Science of Medicine” year 1 course (Fig. 1) [3]. The second session of the program was dedicated to clinical site visits (Fig. 1). Prior to this clinic visit, students were matched to a clinical oncologist who specialized in the disease area; the student had previously identified in the pre-program survey as their particular interest. Our program employed dyads to enhance meaningful learning and foster educational relationships: as such, clinical faculty was paired 1:1 with a given trainee. Subsequently, students were escorted by the program leadership and introduced to their “matched” physician preceptors. Students had the opportunity to participate as an observer with their respective preceptors in up to 6 physician patient encounters. Prior to the encounters, the preceptors were instructed to choose patients that exemplified the impact of discovery science on the diagnosis, molecular classification, genetic analysis, molecular prognostication, or targeted therapeutic approaches used for the treatment/management of the specific malignancy. The final session of the program was devoted to a physician-led debrief, overseen by the TCI Associate Director for Education and Training. During this debrief session, students were asked to reflect on their experiences and consider how the clinical encounter might inform their own research (Fig. 1). During this debrief, notes were transcribed by two of the co-authors. In addition, during the first cohort debrief session, the first author recorded individual participant comments with their permission. Prior to participating in the formal Clinical Encounter Program curriculum, all participants completed an online Health Insurance Portability and Accountability Act (HIPAA) training provided through the Collaborative Institutional Training Initiative (CITI).

Student participation in the TCI Clinical Encounter Program was divided into distinct cohorts, intentionally capped at 5 students per cohort to allow for preceptor: graduate student dyads, to optimize learning. Students selected to participate in each cohort were drawn from the pool of interested students, and reflected diverse student backgrounds, varying years of graduate school training and cancer research interests that overlapped with areas of clinical practice at Mount Sinai. Students in their first year of graduate education were excluded from participation in the TCI Clinical Encounter Program, as they had yet to declare CAB as their major training area. The program was designed to enable successive cohorts of a maximum of 5 students each to engage in the program at distinct and separate periods in time, over the course of the academic year.

### Post-program Evaluation

After engaging in the required debrief session, following their respective clinical encounter experience, graduate student participants were asked to complete an online evaluation survey (Supplementary Table S2) designed to capture the student’s experience. Students were asked if the clinical encounter program had met their expectations, if they felt their experience with the program would have an impact on their PhD training and research, as well as whether they would recommend the clinical encounter program to their colleagues. The survey questions employed were reviewed by CAB and TCI Education leadership prior to utilization in this descriptive evaluation phase of the program. Finally, students were asked to summarize their experiences in a few words and offer recommendations for improvements to the program. These questions were designed to assess post-program attitudes towards clinical encounters in biomedical training and to determine areas of improvement. Survey responses were primarily yes/no, but qualitative data was also captured through open-ended questions. This pilot program, including survey methods utilized, was considered exempt by the Institutional Review Board at the Icahn School of Medicine at Mount Sinai.

## Results

### Pre-program Survey

**Fig. 2:**
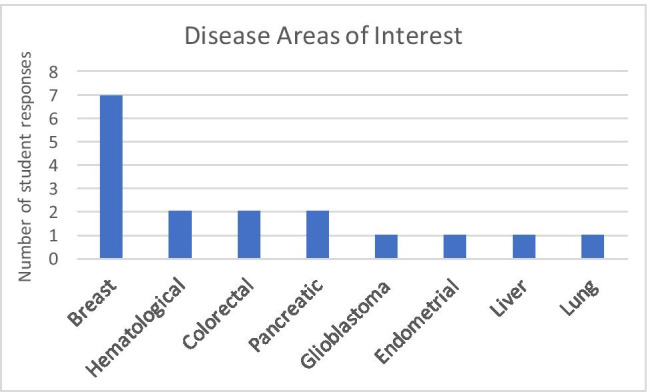
Number of student pre-program survey responses with respect to oncological disease areas of interest plotted by disease type

Out of the 31 PhD students (years 2–5 or more of training) in the CAB Multidisciplinary Training Area that were sent the pre-encounter survey, 17 responded (55%). Survey respondents included students from years 2–5 + of PhD training, but students in their 3rd (*n* = 5) and 4th (*n* = 7) years of training were the most frequent respondents. A total of 11 of the 17 respondents (64.7%) indicated they had not had prior clinical experience, and 15 out of the 17 respondents (88.2%) expressed that exposure to patients with illness would be useful for their training in biomedical sciences. The same number (88.2%) indicated interest in participating in a TCI Clinical Encounter Program pilot. Respondents reported interest in varied neoplastic diseases that present frequently in the ISMMS catchment, including prostate, lung, colorectal, hepatocellular carcinoma, leukemia, and non-Hodgkin lymphoma, though the highest level of interest was in breast cancer (*n* = 7). The pre-program survey results are summarized in Table 1 and Fig. 2.

**Table 1:** Pre-program survey results^*^

Question	Results	
1. Year in the PhD Program?	- Year 2 - Year 3 - Year 4 - Year 5 +	17.7% (*n* = 3) 29.4% (*n* = 5) 41.2% (*n* = 7) 11.8% (*n* = 2)
2. Interested in participating in a clinical exposure program	- Yes	55% (*n* = 17)
3. Exposure to patients is useful for training in biomedical sciences	- Yes	88.2% (*n* = 15)
4. Previously shadowed a clinician, volunteered in a clinic, or spent a significant amount of time in a clinical setting	- Yes	35.3% (*n* = 6)

^*^Percentages from questions 1 and 2 are calculated from both survey responders and non-responders, while percentages from questions 3 to 4 are calculated from only survey responders

### Clinical Encounter

In our needs assessment survey, 15/17 respondents had indicated that they would be interested in participating in a structured clinical experience, if it was offered. In response to this needs assessment, we designed and implemented this pilot TCI Clinical Encounter Program. Thirteen of the original 17 respondents (76%) who had expressed an interest elected to actually participate in the pilot program. Two separate cohorts of 5 students each, for a total of ten students, were selected to participate in this pilot program in 2019. A third cohort, comprised of 3 students, was scheduled to participate during the first quarter of 2020; however, this planned clinical encounter was aborted due to the impact of the COVID-19 pandemic on ambulatory care operations in oncology. As a result, only 10 of the original group of 13 students who expressed an interest in participating in the clinical encounter program were able to engage in this initial feasibility pilot.

On two separate iterations of the clinical encounter experience, cohorts of five students were guided to the clinical site where they would be seeing patients, and introduced to their faculty preceptor. Students, paired with respective oncology experts, participated as observers in disease-focused practices (Leukemia, Lymphoma, Melanoma, Gastrointestinal Cancer and Breast Cancer) in the Ruttenberg Cancer Treatment Center and Breast Cancer and Dubin Breast Center, respectively. Trainees also had the opportunity to interact with members of the patient’s healthcare team, including clinical postdoctoral fellows, nurses, medical assistants, physician extenders, and social workers, thereby enabling trainees to gain insight into the team-based approach fundamental to oncology clinical care.

### Debrief Session

During the faculty-facilitated debrief session, each complete cohort of students provided feedback, shared observations, and expressed their feelings concerning their clinical experience. When the students were asked to describe the single most important thing they took away from their experience in the clinical exposure program, they highlighted the resilience of patients, the importance of research in clinical decision making, and the command of the basic science and clinical literature demonstrated by the physician preceptors. As one student stated:

I was really struck by the art of practicing medicine. I think we tend to believe in science that things are black and white, and seeing the clinicians make decisions when there was no clear right or wrong answer was fascinating. On a personal note, seeing the resilience of the patients in the face of terminal illness was truly inspiring. It reminded me of why I started biomedical research in the first place and has reignited my passion for my research.

Other students provided the following commentary:

It allowed me to learn more about unmet needs in oncology research and at the same time gave a sense of meaning to the research we do in the lab.

It far exceeded my expectations. Especially as someone who had a significant amount of clinical exposure prior to my PhD, I thought that this would be a nice experience but nothing too new for me. I was completely wrong. Being in the clinic after my PhD training this far was so impactful.

When the students were asked to suggest ways to improve the pilot program, students mentioned that there was no appropriate time during the clinical encounter to deliver the one-liner they had practiced during their group exercise on the first day of the program, and recommended additional experiences to have conversations with cancer patients or survivors about their research.

### Post-program Evaluation

Out of the 10 students who participated in the TCI Clinical Encounter pilot Program, 5 responded (50%) to the post-program survey sent immediately following the completion of the program, 3 from the first cohort of 5 students, and 2 from the second cohort (Table 2). While a number of students who participated in the program did not respond to the post-program survey, raising concerns for selection bias, those who did were positive in their feedback and supportive of the program continuing. Four out of 5 respondents felt that the TCI Clinical Encounter Program met their expectations and that their experience in the clinical exposure program would impact their PhD training. Notably, all 5 respondents indicated that they would recommend the program to their colleagues, and 2 of the students contacted the program directors to ask if they could participate again the following year.

**Table 2:** Post-program survey results^1^

Question	Results*	
1. TCI Clinical Encounter Program met expectations	- Yes	80% (*n* = 4)
2. TCI Clinical Encounter Program impacted training in biomedical sciences	- Yes	80% (*n* = 4)
3. Would recommend TCI Clinical Encounter Program to colleagues	- Yes	100% (*n* = 5)

^1^Based on 5/10 (50%) responders

^*^Results are expressed as percentages of survey responders, for questions 1–3

Although we did not incorporate a plan to survey faculty to gain insight into their experience in this pilot, informal conversations with all participating faculty revealed considerable enthusiasm for the program which they found rewarding and a willingness to continue to contribute to this type of clinical encounter experience, in the future.

## Discussion

The development and implementation of the TCI Clinical Encounter Program, designed to address an identified educational gap and unmet need for CAB pre-doctoral trainees at ISMMS and the Graduate School of Biomedical Sciences (GSBS), proved to be feasible. Fifty-five percent (17/31) of CAB students responded to the initial needs assessment survey. Eighty-eight percent of respondents (15/17) reported interest in a structured clinical experience, indicating that even in a cancer biology basic science program, students may be curious to learn about how basic science research in cancer informs clinical oncology. Thirteen of the 17 respondents (76%) elected to enroll in this pilot program, reflective of a demonstrated interest in this curricular activity. Finally, our post-program evaluation revealed that 5/5 (100%) of pre-doctoral CAB trainees who participated in this pilot initiative would recommend the TCI Clinical Encounter Program to colleagues.

While our response rate for our pre-program single request online needs assessment survey was more than expected, based on prior literature for these types of surveys, we acknowledge the limitations of these survey findings given the potential for selection bias [4]. Nevertheless, this data provided insight into participant interest in a potential opportunity to inform the cancer research perspective of emerging PhD scientists in cancer biology.

In developing this program, we sought CAB leadership input and endorsement so as to position this program as a worthwhile endeavor for students to electively pursue. In an effort to harmonize intentions, optimize alignment of expectations, and facilitate meaningful crosstalk between clinicians and pre-doctoral cancer researchers, we also were intentional in our instructions for both clinical scientists serving as trainee mentors and students alike, to demonstrate the integration of scientific discovery at the bedside. Challenges to implementation centered on the logistics and ability to schedule student clinical site visits to accommodate both clinical practice availability and the competing research needs of participating students involved in ongoing laboratory research. Designating both a student and faculty liaison to facilitate student and faculty introductions and to chaperone students to their respective clinical sites was essential for minimizing delays and fostering a smooth transition into the clinical arena.

Students reported the absence of an appropriate time during their clinic visits to present the research one-liner they had practiced in the first program session. In future iterations of the program, students suggested including a separate informal research-symposium geared towards presenting student work to cancer survivors treated at Mount Sinai. This would provide emerging cancer researchers the opportunity to convey their science in understandable terms to an interested and vested lay audience.

Clinical experiences for PhD students in Cancer Biology not only illuminate knowledge gaps in medical practice that can be addressed by experiments in the lab, but also provide insight into the lived experiences of cancer patients and the clinicians who care for them. Our post-program qualitative assessment highlighted the inspiration students derived from this experience, to further fuel their research. Given the recent evidence of burnout among pre-doctoral students in their 4th and 5th years of training, experiences, such as this pilot initiative, that immerse students in the clinical practice have the potential to be strongly motivational [5, 6].

In 2008, CABTRAC published guidelines for PhD education in oncological sciences, focusing on programs to facilitate crosstalk between clinical and basic science disciplines in major cancer research centers [7]. These guidelines were updated in 2015 and include clinical exposure and shadowing programs for PhD students, yet less than a third of students in a 2017 survey of CABTRAC trainees responded that they have structured clinical experience programming in their institutions [8]. The notable discrepancy between the educational community’s consensus and the actual educational practice highlights the need for structured clinical encounter programming for PhD students. To our knowledge, this program is the first example of an oncology-specific, structured clinical encounter program for graduate-level students pursuing a PhD in cancer biology.

Based upon the continued interest of both students and faculty for this pilot initiative, we plan to create a formal elective in the GSBS to advance, scale, and formally evaluate the impact of this structured clinical exposure on graduate-level cancer biology research education. This clinical exposure programs may also be beneficial to a broader community of cancer biology postdoctoral trainees and additional graduate students from other multidisciplinary training areas (MTAs) in the GSBS, who are also pursuing research in cancer. In this regard, the TCI postdoctoral cancer research community has already requested to participate in the TCI Clinical Encounter Program, to further inform and advance their research endeavors.

The initial goal of this program was to increase awareness of disease specific cancers, to further inspire the basic research agenda, and to provide insight into how discovery science ultimately impacts care. An unintended consequence of this pilot has been an evolution of our thinking to include future assignment of clinical mentors to PhD in cancer biology students to provide a window into the clinical realm to further inform their research.

Our educational pilot reveals that a cancer patient TCI Clinical Encounter Program is both of interest to students in our graduate-level cancer biology program and feasible. As such, other cancer biology PhD programs may consider developing and testing similar models to advance an additionally informed cancer research perspective. Future efforts to delineate and describe similar educational experiences for PhD in cancer biology programs nationally may provide added insight into the utility of this type of integrative educational approach to advance doctoral cancer research training.

## Supplementary Information

Below is the link to the electronic supplementary material.

Supplementary file1 (DOCX 20 KB)

## Data Availability

Not applicable.
