# Supplementary material for: The TCI Clinical Encounter Program for PhD Students in Cancer Biology: a Feasibility Pilot
Source: J Cancer Educ. 2021 Oct 14;38(1):134–40. doi: 10.1007/s13187-021-02088-x (PMC8516331; doi:10.1007/s13187-021-02088-x)
Supplement: Supplementary file 1 — Supplementary file1 (DOCX 20 KB) [file 13187_2021_2088_MOESM1_ESM.docx]

**Supplementary Table S1.** Pre-Program Survey Questions

| **QUESTION** | **ANSWER CHOICES** |
| --- | --- |
| **1. (Multiple Choice)**  Would you be interested in participating in a clinical exposure program designed to introduce PhD students to clinicians and patients at Sinai? | - Yes  - No |
| **2. (Long Answer)**  If you have one or more disease areas of interest pertinent to your research, please list them here (i.e., Breast Cancer, Melanoma etc.) | Open text |
| **3. (Long Answer)**  If you have one or more biological areas of interest pertinent to your research please describe them in a few words. (i.e. Genetics, Pharmacology etc.) | Open text |
| **4. (Multiple Choice)**  Do you feel that exposure to patients with illness would be useful for your training in biomedical sciences? | - Yes  - No |
| **5. (Multiple Choice)**  Have you ever previously shadowed a clinician, volunteered in a clinic, or spent a significant amount of time in a clinical setting for other reasons? | - Yes  - No |
| **6. (Long Answer)**  If you answered yes to question 5, please describe briefly. If you answered no, please leave blank: | Open text |
| **7. (Multiple Choice)**  What year are you currently in the PhD Program? | - Year 1  - Year 2  - Year 3  - Year 4  - Year 5+ |
| **8. (Long Answer)**  What are your expectations for this clinical exposure program? Please describe in a few words: | Open text |

**Supplementary Table S2.** Post-Program Survey Questions

| **QUESTION** | **ANSWER CHOICES** |
| --- | --- |
| **1. (Long Answer)**  Did the clinical exposure program meet your expectations? Please describe why yes or no in a few words: | Open text |
| **2. (Multiple Choice)**  Do you believe your experience with the clinical exposure will have an impact on your PhD training and research? | - Yes  - No |
| **3. (Long Answer)**  In a few words, what was the most important thing you took away from your clinical exposure program experience? | Open text |
| **4. (Multiple Choice)**  Would you recommend the clinical exposure program to your colleagues or classmates? | - Yes  - No |
| **5. (Long Answer)**  What would you suggest to improve the clinical exposure program? | Open text |
